# Comparative analysis of bacterial populations in sulfonylurea-sensitive and -resistant weeds: insights into community composition and catabolic gene dynamics

**DOI:** 10.1007/s11356-024-34593-z

**Published:** 2024-08-16

**Authors:** Jan Homa, Wiktoria Wilms, Katarzyna Marcinkowska, Paweł Cyplik, Łukasz Ławniczak, Marta Woźniak-Karczewska, Michał Niemczak, Łukasz Chrzanowski

**Affiliations:** 1 Department of Chemical Technology, Poznan University of Technology, 60-965 Poznan, Poland; 2 Department of Weed Science, Institute of Plant Protection – National Research Institute, 60-318 Poznan, Poland; 3 Department of Food Technology of Plant Origin, Poznan University of Life Sciences, 60-624 Poznan, Poland

**Keywords:** Sulfonylurea, Ionic liquids, HILs, Herbicide, *Centaurea cyanus* L., Biodiversity

## Abstract

This study aimed to compare the impact of iodosulfuron-methyl-sodium and an iodosulfuron-based herbicidal ionic liquid (HIL) on the microbiomes constituting the epiphytes and endophytes of cornflower (*Centaurea cyanus* L.). The experiment involved biotypes of cornflower susceptible and resistant to acetolactate synthase inhibition, examining potential bacterial involvement in sulfonylurea herbicide detoxification. We focused on microbial communities present on the surface and in the plant tissues of roots and shoots. The research included the synthesis and physicochemical analysis of a novel HIL, evaluation of shifts in bacterial community composition, analysis of the presence of catabolic genes associated with sulfonylurea herbicide degradation and determination of their abundance in all experimental variants. Overall, for the susceptible biotype, the biodiversity of the root microbiome was higher compared to shoot microbiome; however, both decreased notably after herbicide or HIL applications. The herbicide-resistant biotype showed lower degree of biodiversity changes, but shifts in community composition occurred, particularly in case of HIL treatment.

**Graphical Abstract:**

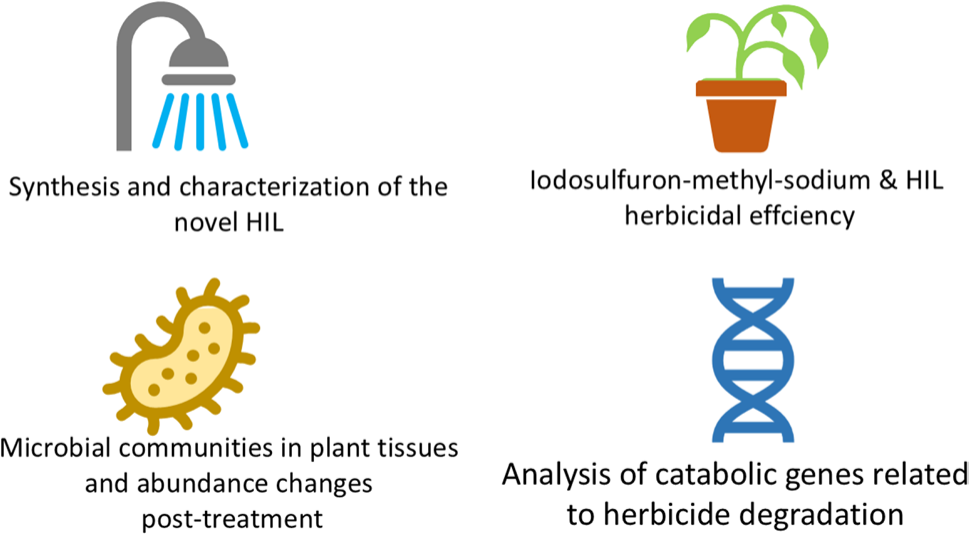

**Supplementary Information:**

The online version contains supplementary material available at 10.1007/s11356-024-34593-z.

## Introduction

Sulfonylurea-based herbicides are widely used in agriculture to limit the growth of monocotyledonous and dicotyledonous weeds (Barros et al. 2016). Such herbicides act via blocking the operation of acetolactate synthase (ALS), an enzyme responsible for the synthesis of branched amino acids (BCAA), such as leucine, valine and isoleucine (Forouzesh et al. 2015; Heap, n.d.; Rosario et al. 2011). Furthermore, this enzyme is only present in both plant and bacterial cells, which limits the toxicity of sulfonylurea-based compounds to animals and people (Chipman et al. 1998; Whitcomb 1999). The sulfonylurea herbicides effectively control weeds using low doses of active ingredient of several grams per hectare (Sarmah and Sabadie 2002). Consequently, sulfonylurea herbicides are very popular among farmers and constitute one of the most popular herbicide classes in the world (United Nations Food and Agriculture Organisation, n.d.). The persistence of this group of compounds in the environment strongly depends on soil pH values, with half-life values spanning from weeks in acidic soil up to years under alkaline conditions (Lei et al. 2023b). For example, the half-lives of nicosulfuron shift from 15 to 20 days in case of acidic pH to 190–250 days in case of neutral/alkaline pH (Zhong et al. 2023). In the latter case, the residual herbicides may migrate to groundwater and surface water, posing a threat to aquatic plants, non-target crops, aquatic animals as well as humans (Zhong et al. 2023). It has been confirmed that exposure to nicosulfuron can induce morphological and behavioural changes in spined toad tadpoles (Cheron et al. 2023) as well as induce hyperglycemia, increase the risks of cardiovascular diseases and lead to denaturation of serum albumin in humans (Zhong et al. 2023). Moreover, the presence of sulfonylurea herbicides may alter the enzymatic activity soil microorganisms as decreased activity of dehydrogenases and phosphatases upon exposure has been reported (Medo et al. 2020). In addition to these issues, one of the most challenging problems is the rapid rate of global development of weed resistance to this group of herbicides (Heap, n.d.). The spread of weed resistance to applied herbicides is a major issue in worldwide crop protection (Lei et al. 2023b). Currently, 272 species of weeds have evolved herbicide-resistant biotypes in 100 crop plants across 72 countries (Heap, n.d.; Marcinkowska et al. 2023; Zhong et al. 2023). To date, cornflower biotypes resistant to herbicides have only been confirmed in Poland (Heap, n.d.).

Adjuvants are widely used in all herbicidal mixtures to improve the properties of the final formulation (Aparecida et al. 2013; Mesnage et al. 2014, 2013; Wilms et al. 2020b). Recently, concerns regarding high toxicity of some additives have been raised, e.g., in the case of ethoxylated etheralkylamine or solvent naphtha (Defarge et al. 2018; Mesnage et al. 2013; Mesnage and Antoniou 2018). As a result, herbicidal ionic liquids (HILs) have been proposed in order to limit the use of adjuvants (Pernak et al. 2011; Wilms et al. 2020b). This class of compounds combines the herbicidal activity of typical formulations with improved surface active properties in a single compound, thus allowing for simplification of herbicidal formulations for field use (Niemczak et al. 2019, 2017; Pernak et al. 2016, 2011; Tang et al. 2020, 2018; Wang et al. 2019; Wilms et al. 2020b). Another potential benefit of transforming herbicides into an ionic liquid form is the great potential of adjusting physicochemical properties of such compounds via selection of cations and ease of their modifications, which also allows to fine tune biological properties (Wilms et al. 2020b). Nevertheless, most of the currently available research regarding HILs is focused on synthesis, physicochemical properties and determination of herbicidal activity towards weeds and crop plants (Wilms et al. 2020b). Available studies regarding the microbiome rarely traverse beyond toxicity tests of HILs toward model microorganisms or biodegradation studies (Wilms et al. 2020b); however, recently this sentiment is slowly changing and more insight into HILs interactions with non-target organisms is being provided (Parus et al. 2023, 2021; Stachowiak et al. 2021; Wilms et al. 2023a, b; Woźniak-Karczewska et al. 2022). This is especially important in the case of HILs based on sulfonylurea herbicides, as negative impacts of sole sulfonylurea herbicides on the environment as well as metabolic pathways utilised by the bacteria in order to dissipate such xentobiotics have been well evidenced (Lei et al. 2023a; Zhong et al. 2023).

It has already been reported that the cation and anion in HILs are degraded at different rates, which may indicate that ionic liquids are rather an application form of the herbicide than an entity that is stable in the environment (Wilms et al. 2023b, a). As evidenced by Wilms et al. (2023a, b), sorption is a factor significantly decreasing the bioavailability of cations in soil, which may result in their bioaccumulation and limited degradation, and potentially negatively affect the biotransformation of herbicidal anions (Wilms et al. 2023a, b). These findings, which put the integrity of HILs under question, are supported by the research of Woźniak-Karczewska et al. (2022), which demonstrated that the cation and anion adsorption parameters of 2,4-D HILs were completely independent and the cations’ adsorption *K*_*f*_ values were correlated with its hydrophobicity (Woźniak-Karczewska et al. 2022). Furthermore, studies investigating soil microcosm exposed to HILs reveal that the cation selection is the determining factor in microbiome community composition changes (Wilms et al. 2023b; Woźniak-Karczewska et al. 2022). As was demonstrated by the abovementioned studies, the form of the HIL and structure of the cation in particular have significant implications for environmental behaviour of such xenobiotics.

To summarize, although the overall awareness regarding the environmental impact of sulfonylurea compounds has been steadily increasing during recent years, the number of studies which evaluate the in-depth impact of such herbicides on the diversity of the soil microbiome is limited; among such reports, experiment are mainly focused on nicosulfuron, while data regarding the impact of other sulfonylureas is scarce; comparison of classic herbicides and HILs usually revolves around their efficacy in fighting weeds, with limited insight into environmental impacts, which notably restricts the possibility to conduct a proper risk assessment; finally, some studies have hinted that HILs may be an effective tools to control herbicide-resistant weed species (Marcinkowska et al. 2023; Pernak et al. 2022); however, no insightful rationale which would elucidate this phenomenon has been presented to date.

The importance of this study is associated with an attempt to tackle the abovementioned knowledge gaps. The aim was to evaluate the effect of iodosulfuron-methyl-sodium (selected as a model herbicide for effective weed control) and HILs based on the same active ingredient on the microbiome associated with cornflower, a commonly occurring weed in field crops. The novelty of the described findings results from the fact that, for the first time, the impact of herbicides and corresponding HILs on herbicide-resistant weeds was evaluated at the genetic level. We assumed that the microbiome present on the surface of the plant and in its tissues may aid the weeds in deactivation of active ingredient in the spray solution. In order to verify this hypothesis, we investigated microbiome diversity of susceptible (*S*) and resistant (*R*) biotypes of cornflower and assessed the presence and abundance of genes responsible for degrading sulfonylurea-based herbicides in the soil, rhizosphere and among the plants’ epiphytes and endophytes. All of the currently known genes that participate in the degradation of sulfonylurea herbicides were selected for this purpose. The obtained data may be valuable for defining the scope of risk assessment necessary for registering HILs as commercial agrochemicals, which contributes to its high significance.

## Materials and methods

### Chemical reagents

The following reagents were used during the experiments: iodosulfuron-methyl sodium salt (96.6%, PESTINOVA, Jaworzno, Poland), 2-dimethylaminoethanol (≥ 99.5%, Sigma-Aldrich, Saint Louis, MO, USA) and 1-bromotetradecane (97.0%, Sigma-Aldrich, Saint Louis, MO, USA), methanol, acetonitrile, chloroform (Avantor, Gliwice, Poland) and deionized water (conductivity < 0.1 μS·cm − 1, obtained using a HLP Smart 1000 demineralizer, Poznań, Poland).

### Synthesis and analysis of HILs

#### Synthesis of *N*-tetradecylcholine bromide

A quaternization reaction was used to synthesize *N*-tetradecylcholine bromide based on the following steps: (i) 30 mL of acetonitrile were added to a 100-mL round-bottom flask, followed by 2-dimethylaminoethanol (0.05 mol) and 1-bromotetradecane (0.0505 mol); (ii) the flask was equipped with a Teflon-coated magnetic stirring bar; (iii) all the reactants were subjected to stirring for 48 h at 60 °C; (iv) evaporation of the solvent was carried out using a vacuum evaporator; (v) the residues were treated with ethyl acetate (100 mL) which allowed to precipitate the product as a white solid; (vi) after cooling the solution to 5 °C, separation of the product was carefully conducted using vacuum filtration with a glass filter funnel; (vii) finally, the isolated filtrate was washed using cooled ethyl acetate and subjected to drying under reduced pressure at 50 °C for 24 h.

#### Synthesis of the herbicidal ionic liquid

In order to obtain the actual herbicidal ionic liquid, an ion exchange reaction was conducted with the use of an Easy-Max reactor based on a procedure described in a previous study (Niemczak et al. 2020). The general outline of the reaction included the following steps: (i) methanol (15 mL) was introduced into a 100-mL reaction vessel and used to dissolve *N*-tetradecylcholine bromide (0.01 mol); (ii) sodium salt of iodosulfuron-methyl (0.0102 mol) dissolved in methanol (15 mL) was added to the system in order to conduct the ion exchange; (iii) the reaction vessel was equipped with a mechanical stirrer, the system was stirred at 50 °C for 15 min and subsequently cooled to 0 °C; (iv) the inorganic by-product (NaBr) was removed via filtration and then the solvent was removed via evaporation; (v) the residue of the crude product was dissolved in chloroform (15 mL) in order to remove any impurities; (vi) the purified product was separated via filtration and residual chloroform was evaporated; (vii) in the last step, the product was dried under reduced pressure at 50 °C for 24 h and stored over P_4_O_10_.

#### Spectral analysis

In order to confirm the structure of the final product and analyse its purity, ^1^H and ^13^C NMR spectra was well as IR spectra were obtained and analysed. Varian VNMR-S 400 MHz spectrometer was used to obtain the NMR spectra, with operating frequency at 400 MHz for ^1^H NMR (with the tetramethylsilane as the internal standard) and 100 MHz for ^13^C NMR, respectively. In case of IR spectra, the semi-automated system EasyMax 102 (Mettler Toledo, Switzerland) with a ReactIR iC15 probe was used to obtain spectral data, which was processed using the iCIR 4.3 software.

#### Water content

Karl Fischer titration method, which is a standard way to determine water content in synthesised HILs (Niemczak et al. 2019; Niu et al. 2018; Stachowiak et al. 2022; Tang et al. 2018; Wilms et al. 2020a), was employed in order to evaluate the water content in all the samples, with the use of a TitroLine 7500 KF trace apparatus (SI Analytics, Germany). The procedure is based on the following steps: (i) samples were dissolved in dehydrated methanol; (ii) the concentration of water in pure methanol as well as the sample solutions was determined based on titration; (iii) the water content in pure products was established based on the difference between results obtained in step (ii).

#### Melting point

The MP 90 melting point system (Mettler Toledo, Switzerland) calibrated based on certified reference substances was used to measure the melting points of all the obtained compounds.

### Plant material and method of weed control

Evaluation of herbicidal efficacy was carried out with the use of susceptible and herbicide-resistant cultivars of cornflower (*Centaurea cyanus* L.) at the Institute of Plant Protection – National Research Institute. Previous tests carried out with the resistant cultivar confirmed that it exhibits resistance to herbicides from the group of ALS inhibitors. The respective resistance index (RI) classified using Beckie and Tardif’s scale modified for ALS inhibitors (Beckie and Tardif 2012) exceeded the value of 71.4, which corresponds to very high resistance (Burgos 2015).

In order to conduct the experiment, pots with a commercial, acidic medium (Kronen, Cerekwica, Poland) were used to sow cornflower seeds (depth of 1 cm), which were subsequently kept in a greenhouse under controlled conditions (temperature at 20 ± 2 °C, air humidity at 60%, photoperiod of 16/8 h day/night). Appropriate soil moisture in the pots was ensured by watering. After 16 days, the number of seedlings per pot was thinned to four. Treatment with the studied herbicidal ionic liquid was carried out at the 4-leaf stage (BBCH 14), along with a commercial herbicide formulation Autumn 10 WG (10% iodosulfuron-methyl-sodium; Bayer, Germany) which served as a reference. The doses of both HIL and the reference herbicide were equal to 10 g of iodosulfuron-methyl sodium salt per ha (calculated with regard to the active substance). A moving nozzle sprayer equipped with flat-fan TeeJet 110/02 VP nozzle (TeeJet Technologies, Wheaton, IL, USA) which delivers 200 L/ha of spray solution at an operating pressure of 0.2 MPa and constant speed of 3.1 m/s was used to carry out the treatment. The spraying system was placed at a distance of 40 cm above the plants. After treatment, the respective sample series were placed in the greenhouse again to ensure controlled environmental conditions. The experiment was terminated after 3 weeks. After this period, both-treated samples and non-treated controls were disassembled into three respective sub-samples which included the above-ground part of the plants, their roots and soil from the vicinity of plants and their rhizosphere, which were collected for subsequent analyses.

### Isolation of microbial communities from greenhouse samples

#### Isolation of microorganisms from the soil and plants’ rhizosphere

In order to conduct the isolation of soil microorganisms, soil samples (2 g) collected as described in the previous step were homogenised, introduced into sterile Erlenmeyer flasks (150 mL) and subjected to an isolation procedure described by Hallmann et al*.* (2017) (Hallmann et al. 2007). In general, the soil was inoculated with a 50% TSB medium (25 mL) and incubated at 30 °C for 48 h on a rotary shaker (120 rpm). Subsequently, after decantation of the medium from soil, the microbial cells were separated via centrifugation (4500 rpm, 4 °C for 15 min) and stored using glycerol stocks (20% v/v, − 80 °C). All samples were prepared with three repetitions.

#### Isolation of epiphytes from plant tissues

For isolation of epiphytes, the plant material was washed under tap water to remove soil residues for approx. 10–15 min (Anjum and Chandra 2015). After washing, 1 g of the respective plant material (either leaves and shoots or roots) was separated and introduced into a sterile beaker under a laminar flow cabinet. Afterwards, sterile deionized water was added and the content was stirred for 1 min. Subsequently, 1 mL of the leachate after washing was transferred into a sterile Erlenmeyer flask (150 mL) using a pipette, followed by inoculation with a 50% TSB medium (25 ml) and incubation at 30 °C for 48 h on a rotary shaker (120 rpm). Finally, the microbial cells were centrifuged (15 min, 4500 rpm, 4 °C) and stored in glycerol stocks (20% v/v, − 80 °C), in accordance with section ‘Isolation of microorganisms from the soil and plants’ rhizosphere’. All samples were prepared with three repetitions.

#### Isolation of endophytes from plant tissues

In case of isolation of endophytes, the plant material was washed, then drained from excess water and cut into three equal parts (approx. 0.3 g each). The parts were introduced into a sterile beaker under a laminar flow cabinet. To extract the endophytes, the plant material was subjected to treatment with 70% ethanol for 30 s, followed by sodium hypochlorite solution (2%, v/v, with 2 mL/L of Triton X-100 surfactant) for 2 min, and 70% ethanol again for 30 s. The samples were then rinsed with deionized water five times, then ground under the laminar flow cabinet using a sterile mortar. Subsequently, 1 mL of the ground plant material was transferred into a sterile Erlenmeyer flask (150 mL), followed by inoculation with a 50% TSB medium (25 mL) and incubation at 30 °C for 72 h on a rotary shaker (120 rpm). Finally, the microbial cells were centrifuged (15 min, 4500 rpm, 4 °C) and stored in glycerol stocks (20% v/v, − 80 °C), in accordance with section ‘Isolation of microorganisms from the soil and plants’ rhizosphere’. All samples were prepared with triple repetitions.

### Identification of microbial community composition via 16 s RNA sequencing

#### Isolation of DNA for 16s RNA sequencing

DNA was extracted using the Genomic Mini Spin kit (060-100S, A&A Biotechnology, Gdańsk, Poland) in accordance with the protocol provided by the manufacturer. After elution of the purified DNA, the isolates were stored at − 80 °C after neutralization to ensure minimal degradation of the matrix.

Evaluation of isolation efficiency was based on a fluorometric analysis with the use of Qbit 3.0 device and the Qubit™ dsDNA HS Assay Kit (Q32851, ThermoFisher Scientific, Waltham, MA, USA). In case of each sample, three independent DNA extractions were carried out and the material was combined following positive quantification.

#### PCR amplification and NGS sequencing

PCR and NGS sequencing are commonly employed methods when it comes to analysis of xenobiotics impact on soil microbiome, that is able to accurately demonstrate shifts in composition of bacterial community (Huang et al. 2023; Pang et al. 2023; Wilms et al. 2023b, a). Therefore, we have decided to utilize similar approach in this study. Briefly, PCR was conducted using the Ion 16S™ Metagenomics Kit (A26216, Life Technologies, Carlsbad, CA, USA) in accordance with the protocol provided by the manufacturer. The kit is designed to amplify the V2–V9 regions of the bacterial 16S rRNA gene. The reaction consisted of 15 µL of 2 × Environmental Master Mix, 3 µL of the appropriate primer and 12 µL of the previously isolated DNA sample and was carried out using the VeritiPro thermal cycler (Life Technologies, Carlsbad, CA, USA). The following temperature program was applied: initial denaturation for 10 min at 95 °C; 25 cycles of denaturation for 30 s at 95 °C; annealing for 30 s at 58 °C; extension for 20 s at 72 °C; and a final extension for 7 min at 72 °C.

The products of the PCR reaction were purified with the use of the Agencourt AMPure XP Reagent (A63880, Beckman Coulter, Pasadena, CA, USA) in accordance with the protocol provided by the manufacturer. The DNA was bound to magnetic beads and contaminants were purified using ethanol. Afterwards, the DNA was eluted with the use of nuclease-free water or low-TE buffer. Subsequently, a library was prepared and purified using Agencourt AMPure XP Reagent (A63880, Beckman Coulter, Pasadena, CA, USA) in accordance with the instructions of the Ion Plus Fragment Library Kit (4,471,252, Life Technologies, Carlsbad, CA, USA). Upon isolation, the DNA was subjected to storage. Determination of library concentration was conducted with the use of Ion Universal Library Quantitation Kit and Quant Studio 5 real-time PCR instrument (A26217, Life Technologies, Carlsbad, CA, USA). In the next step, the library concentration was diluted to 10 pM and onto beads in emulsion PCR was carried out using the Ion PGM™ Hi-Q™ View OT2 Kit reagent kit and an Ion One Touch 2 Instrument (A29900, Life Technologies, Carlsbad, CA, USA). The beads were then purified with the use of an Ion One Touch ES Instrument (Life Technologies, Carlsbad, CA, USA) and subjected to sequencing using the Ion PGM™ Hi-Q™ View Sequencing Kit (A29900) on an Ion 316™ Chip Kit v2 BC (Life Technologies, Carlsbad, CA, USA).

#### Bioinformatic analysis

Reads of sequences originating from Ion Torrent (Thermo Fisher Scientific, Waltham, MA, USA) were obtained in the BAM format and imported into the CLC Genomics Workbench 20.0 software (Qiagen, Hilden, Germany). The data was then processed with the use of CLC Microbial Genomics Module 20.1.1 (Qiagen, Hilden, Germany). All chimeric and low-quality reads were removed (based on a quality limit of 0.05 and an ambiguous limit of ‘N’), while the remaining sequences were clustered against the SILVA v119 database. A 97% similarity threshold was used for operational taxonomic units (OTU), and statistically significant differences were evaluated using one-way ANOVA.

### DNA isolation and storage

Isolation of DNA was conducted using the Genomic Mini kit (A&A Biotechnology, Gdańsk, Poland) in accordance with the protocol provided by the manufacturer. In order to confirm the purity and quantity of the isolated DNA, a Qubit 4 Fluorometer and Qubit™ dsDNA HS Assay Kit (Q32851, Thermofisher Scientific, Waltham, MA, USA) was used. Upon isolation, the obtained genetic material was stored in a 0.1 M Tris buffer at − 20 °C for no longer than 2 weeks.

### Determination of genes responsible for degradation of sulfonylurea herbicides

#### Genes and primer sequences for PCR analysis

All genes and corresponding primer sequences used for the analysis were presented in Table S1.

#### Microbial material

*Hanshlegiella zhihuiae* S113 (DSM 18984), *Streptomyces griseolus* 14,576–4 (*Streptomyces halstedii* DSM 40854) and *Bacillus subtilis* 168 (DSM 23778) were used as positive control to ensure proper optimization of PCR reaction parameters. All the strains were obtained for Leibniz Institute DSMZ-German Collection of Microorganisms and Cell Cultures and cultivated in accordance with the provided protocols.

#### PCR reaction

After performing a temperature optimisation for all the primers and ensuring that each primer produced only the main product, appropriate annealing temperatures were established, which were presented in Table S2. Furthermore, the genes were tested using negative templates and no visible products were observed on electrophoretic gel under the temperatures listed in Table S2. In each case, DNA originating from a respective biological sample (25 ng) was used as a template under the following reaction conditions: initial denaturation (1 × ; 4 min at 95 °C), 40 steps of denaturation (30 s at 95 °C), primer annealing (45 s at temperature listed in Table S2) and extension (1 min 45 s at 72 °C), followed by final extension step (5 min at 72 °C) and storage step (∞ min at 4 °C). Control samples with sterile deionized water used as template were also tested.

#### Electrophoresis parameters

In order to separate the PCR reaction products, a 1.5% agarose gel (Prona Agarose; Basica Le, Burgos, Spain) stained with Midori Green Advance DNA Stain (Nippon Genetics Europe, Düren, Germany) for 70 min at 120 V with the use of MultiSub Midi (CleaverScientific, Rugby, UK). Subsequent visualization carried out using a microDOC (CleaverScientific, Rugby, UK) apparatus.

#### Genes and primer sequences for qPCR analysis

Based on previous studies regarding genes abundance in HILs treated soil, the methodology was adapted and utilised in this study (Wilms et al. 2023a, b). Briefly, analysis of gene expression was conducted using the Power SYBR Green PCR Master Mix (Life Technologies, Carlsbad, CA, USA) and ABI 7500 SDS (Applied Bio-systems, Thermo Fischer Scientific, Waltham, MA, USA) in accordance with the protocol provided by the manufacturer. Real-time PCR was carried out with the use of primers listed in Table S3. Amplification of a bacterial 16 S ribosomal RNA fragment with the use of primers and a TaqMan MGB probe was carried out in order to quantify the total bacterial RNA with the use of a TaqMan Universal Master Mix II (Life Technologies, Carlsbad, CA, USA) and ABI 7500 SDS (Applied Biosystems, Thermo Fischer Scientific, Waltham, MA, USA). Each analysis was carried out with three repetitions. The mean expression index was used to evaluate gene expression in each sample, which was calculated based on three analyses using the following formula: *C*_*T*_ target/*C*_*T*_ 16 S. The index represents the level of the specific gene relative to the universal gene (16 S RNA) within the entire metabiome.

### Statistical analysis

The presented results were calculated as average values based on at least four replicates prepared for each sample in a randomized setup, with standard errors of the mean (SEM) for each respective set of samples obtained using the following equation:

$$\text{SEM }=\text{ s}/\text{n}^{0.5}$$

where SEM is the standard error of the mean, *s* is the sample standard deviation and *n* is the number of samples.

The datasets were subjected to one-way ANOVA analysis with *p* < 0.05 in order to determine the statistical significance of evaluated differences and pairwise Kruskal–Wallis tests were conducted for metagenomic data.

## Results and discussion

**Fig. 1:**
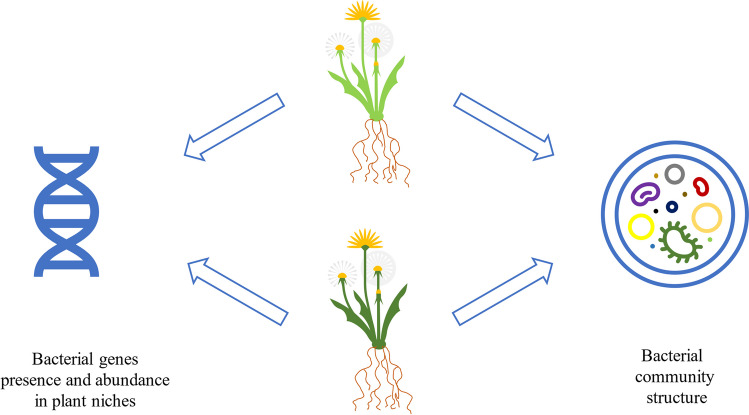
Scheme of molecular analyses performed on susceptible (light green) and resistant (dark green) cultivars of *Centaurea cyanus*

Previous research regarding the environmental effects of ionic liquids with herbicidal activity clearly demonstrated that cation is solely responsible for the toxicity towards the microbiome and the driving force behind structural shifts in the soil microcosms (Stachowiak et al. 2021; Wilms et al. 2023a, b; Woźniak-Karczewska et al. 2022). However, no evidence has been presented so far that similar phenomena occur in the tissues of plants. In this study, we aimed to investigate whether epiphytes and endophytes react in a similar manner compared to soil and rhizosphere microbiome (Fig. 1). Additionally, we selected genes which participate in the biotransformation of sulfonylureas to biologically inactive forms, and we investigated their natural occurrence as well as abundance in isolated environmental samples. This was conducted in order to determine whether HILs had a stronger detrimental effect than the classic form of herbicide on bacteria capable of handling sulfonylurea herbicides, or rather enhanced their proliferation in the microbiome.

Two of the mentioned genes are well established genes from *S. griseolus* 14,576–4 (class: *Actinomycetia*) (Harder et al. 1991; Hussain and Ward 2003; Omer et al. 1990), while additional four novel genes have been discovered recently. Three of them can be found in *B. subtilis* YB 1 (class: *Bacilli*), all involved in the production of extracellular enzymes that possess a secondary function of degrading active form of various sulfonylureas (Kang et al. 2012, 2014; Lu et al. 2012; Z. Zhang et al. 2018a, b; Zhang et al. 2020). The last gene encodes an enzyme from *H. zhihuiae* S113 (class: *Alphaproteobacteria*) with the primal function of degrading sulfonylureas (Hang et al. 2012; Liu et al. 2019); moreover, this gene seems to be expressed on a constant basis (Hang et al. 2012).

Due to the fact that we selected genes that encode extracellular enzymes, we have decided to perform our studies on both susceptible and resistant biotypes of cornflower in order to determine whether the biotype’s resistance is correlated with the composition of its microbiome.

### Synthesis and analysis of HIL

The initial stage of experiments was focused on the synthesis of HIL which was later used for comparative purposes with the commercial herbicide during subsequent studies. The structure of the obtained HIL, its basic physico-chemical properties and synthesis yield are shown in Table 1. The isolated and purified product, obtained with a 92% yield, was a solid at room temperature. Analysis of the melting point revealed that this salt melts at approx. 90 °C, which allows to classify it as an ionic liquid (IL). It was also noted that the product is not hygroscopic, but contains 2.42% of water, clearly indicating that this IL formed a monohydrate (molar ratio equal to 1.09). The obtained NMR spectra are presented in the ESI (Fig. S1-S3).

**Table 1:** Characteristic of synthesized HIL

HIL	Structure	Yield [%]	State at 25 °C	Melting point^a^ [°C]	Water content^**b**^[%]
[C_14_CHOL] [ISM]		92	white solid	89.9–91.5	2.42

^a^Mettler Toledo MP 90 melting point system

^b^SI Analytics TitroLine 7500 KF trace

### Herbicidal efficacy evaluation

In the second stage of studies, the commercial herbicide and HIL (both based on *N*-tetradecylcholine iodosulfuron-methyl as the active compound) were employed to control cornflower biotypes both susceptible and resistant to ALS inhibitors. The tested HIL and commercial product showed herbicidal activity against the susceptible (*S*) cornflower, while they did not effectively control the biotype resistant (*R*) to herbicides from the group of sulfonylureas (Fig. S4). The fresh weight reduction for the susceptible population was 87% for HIL and 94% for the reference product, respectively. In case of the resistant population, both compounds induced mild symptoms of plant damage, with their effectiveness below 15%. Similar to the susceptible samples, a slightly higher efficacy of the herbicide over HIL was observed, although these differences were not statistically significant.

In order to prevent weed resistance to ALS inhibitors (and herbicides in general), it is crucial to use herbicides with different modes of action (Heap, n.d.). Over the past 2 years, two reports regarding ionic liquids comprising two or three active ingredients with distinct modes of action have been released (Marcinkowska et al. 2023; Pernak et al. 2022). In the study by Pernak et al. (2022), HILs containing tribenuron methyl and herbicides from the group of synthetic auxins were tested (Pernak et al. 2022). The obtained results indicate that ionic liquids containing an anion from the phenoxy acid group may limit the development of cornflower resistance to ALS. Additionally, in the work of Marcinkowska et al. (2023), the influence of HILs containing double or triple anions (sulfonylurea and auxin-like herbicides) on weed control of herbicide-resistant cornflower was investigated (Marcinkowska et al. 2023), revealing that the tested compounds efficiently reduced the resistant biotype.

### Impact of HIL on the microbiome

The goal of the third stage of studies was to evaluate the influence of the herbicide and HIL on susceptible and resistant biotypes of cornflower. The description was divided into five subsections, which respectively focus on shifts in the microbiome of the root surface and inner tissues as well as shoot surface and inner tissues, with the final section dedicated to the assessment of biodiversity indices.

#### Root surfaces

The analysis of the root surface microbiome of susceptible or herbicide-resistant cornflower revealed that *Proteobacteria* was the dominant type in all analysed plants, with a relative abundance exceeding 90%, which is consistent with the fact that it is one of the most abundant and diverse types of bacteria present in rhizosphere soil, particularly in the rhizosphere of agricultural crops as well as weeds (Parus et al. 2023). Moreover, many members of this group, such as *Pseudomonas putida*, are well known for their ability to degrade herbicides and other organic xenobiotics (Kivisaar 2020; Poblete-Castro et al. 2012). The presence of specific soil contaminants usually results in proliferation of key players associated with their degradation; thus, a relatively high abundance of herbicide-degrading representatives of *Proteobacteria* can be expected in treated soil. The second most abundant type, *Firmicutes*, ranged from 0.2 to 9.0%, with its lowest relative abundance observed in the root epiphytes of the susceptible cornflower biotype treated with sterile water. Similar abundance levels were noted in samples from both resistant and susceptible biotypes treated with herbicides and the HIL which were comparable to resistant biotype control samples. This finding is notable, given the recognized value of *Firmicutes* in agroecology (Hashmi et al. 2020). Notably, their ability to modulate plant hormonal production or produce analogues of plant hormones could assist plants in coping with herbicidal stress, potentially enhancing their resistance (Hashmi et al. 2020). The contribution of other bacterial types did not exceed 0.05% (Fig. S5). The dominant bacterial classes in this niche were *Gammaproteobacteria*, *Alphaproteobacteria*, *Betaproteobacteria* and *Bacilli*.

#### Root inner tissues

**Fig. 2:**
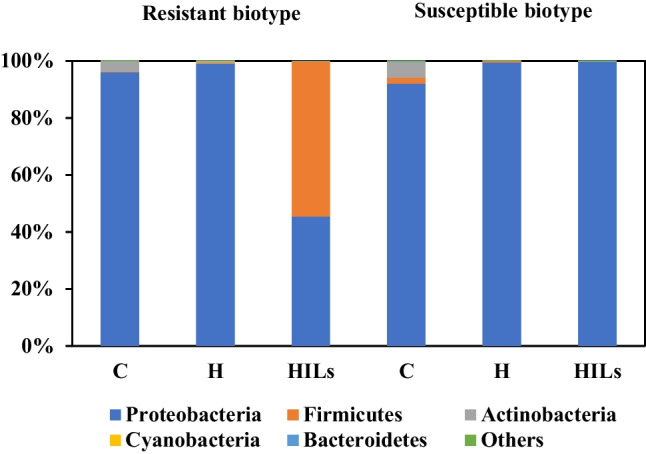
Analysis of the inner root tissue microbiome composition of susceptible and herbicide-resistant cornflower. C, untreated control; H, herbicide treatment; HILs, herbicidal ionic liquid treatment

The analysis of cornflower endophytes, in untreated plants (controls) and plants after herbicide treatment belonging both to resistant and susceptible cultivars, revealed no significant differences in microbiome composition present in the inner tissues of their roots (Fig. 2). In all studied plant tissues, *Proteobacteria* type predominated in controls, contributing from 92 to 99%. Similarly, high levels of *Proteobacteria* were observed in the internal root tissue of herbicide-treated susceptible weeds (99.6%). However, in herbicide-resistant cornflower treated with HIL, the contribution of *Proteobacteria* in endophytes decreased to 45%, with the *Firmicutes* becoming the dominant type at 54%. This phenomenon can be attributed to the fact that the mechanism of action of HILs is dictated by the activity of the cation, and the susceptibility of bacteria is affected by the structure of their cell wall (Parus et al. 2021; Stachowiak et al. 2021; Wilms et al. 2023a, b). Hence, it is probable that endophyte population was more affected by the cation present in the HILs molecule than by the sole herbicide. Untreated plants also exhibited the presence of *Actinobacteria*, ranging from 3.8 to 5.4%, which could not be observed in any of the treated plants. The presence of *Actinobacteria* is considered beneficial in terms of plant growth stimulation as well as plant survival and soil management (Aamir et al. 2019; Singh and Dubey 2018).

At the class level, the dominant ones included *Alphaproteobacteria*, *Gammaproteobacteria*, *Actinobacteria* and *Bacilli*. The increase in the contribution of the *Bacilli* class was particularly prominent in the internal root tissue of plants resistant to herbicides treated with HILs, in case of which they were recorded at 55% of the general population. This contradicts with the previous research conducted by Stachowiak et al. (2021) which demonstrated that *Bacillus cereus* (member of the *Bacilli* class) is generally more sensitive to ionic forms of iodosulphuron-methyl than *P. putida* (member of the *Gammaproteobacteria* class) (Stachowiak et al. 2021).

#### Shoot surfaces

**Fig. 3:**
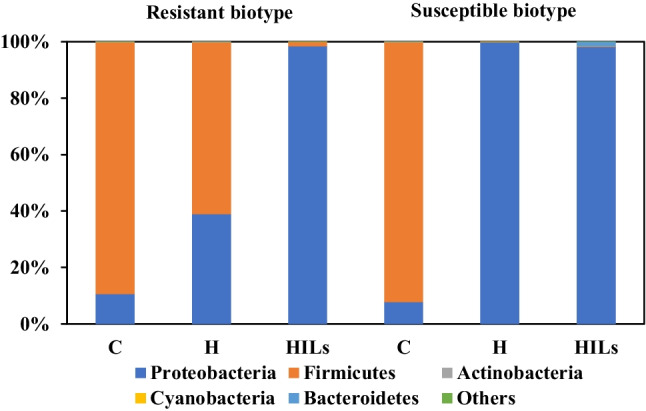
Analysis of the shoot surface microbiome composition of susceptible and herbicide-resistant cornflower. C, untreated control; H, herbicide treatment; HILs, herbicidal ionic liquid treatment

The microbiomes of the shoot surface in both untreated sensitive and resistant biotypes did not differ significantly (Fig. 3). In both cases, the dominant type was *Firmicutes* (90–92.5%). However, subjecting the plants to both herbicide and HIL treatment led to significant changes in the shoot surface microbiome of both plant types. These changes are different in *S* and *R* biotypes, suggesting that the microbiome may contribute to the plant’s resistance to the herbicide in the studied plants. In the case of the resistant populations treated with the sole herbicide, the contribution of *Firmicutes* decreased to 61%, with *Proteobacteria* accounting for the remaining 39%. The decreased abundance of *Firmicutes* contradicts with the previously presented increase for root inner tissues, and indicates a plausible migration of bacteria belonging to this phylum from shoot surface into the roots. On the contrary, *Proteobacteria* dominated the microbiome of the shoot surface of susceptible cornflower treated with the herbicide, constituting 99.7% of the microbiome. This dominance (exceeding 98%) is also evident in both S and R populations after the application of HILs. However, it must be noted that the phyllosphere microbiome exhibits greater dynamism compared to rhizosphere and endosphere environments (Dastogeer et al. 2020). Namely, microbial inhabitants experience variations in heat, moisture and radiation throughout the day and seasons, and the phyllosphere is mainly exposed to herbicidal spraying. Furthermore, these environmental factors impact plant functions such as photosynthesis, respiration and water uptake, thereby indirectly shaping the composition of the microbiome. Overall, these results indicate that in case of resistant biotypes HILs contribute to a significant shift in the structure of the microbiome compared to the reference herbicide, similar to the inner root tissue experiment.

#### Shoot inner tissues

**Fig. 4:**
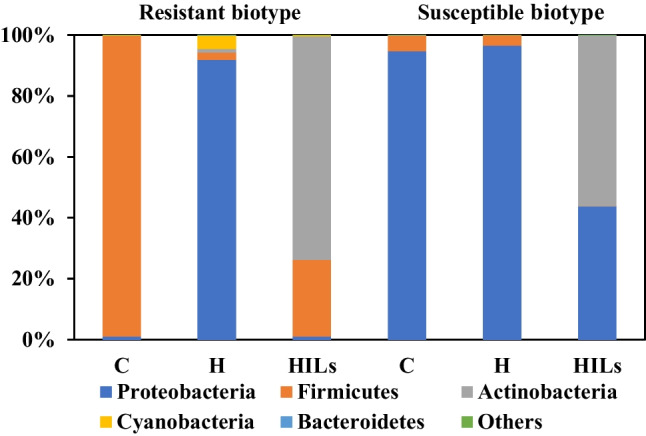
Analysis of the inner shoot tissue composition of microbiome of susceptible and herbicide-resistant cornflower. C, untreated control; H, herbicide treatment; HILs, herbicidal ionic liquid treatment

The analysis of the microbiome in both untreated sensitive and resistant biotypes subjected to treatment with sterile water revealed significant changes in their microbiomes (Fig. 4). Bacteria from the *Firmicutes* type dominated the tissue of the herbicide-resistant plants, specifically the class *Bacilli* (99%), while *Proteobacteria* were dominant (96.5%) in the tissue of herbicide-susceptible plants, with the class *Gammaproteobacteria* accounting for 91% of the microbial composition. The application of the herbicide did not cause changes in the microbiome of the sensitive populations, but in the case of the resistant populations, the bacterial community structure shifted significantly and resembled that of herbicide susceptible biotypes, albeit *Cyanobacteria* were only detected in *R* biotypes. This finding further suggest that, in the examined plants, the microbiome may be partially responsible for the weeds’ resistance to herbicides, as *Cyanobacteria* are known to improve plants’ stress tolerance (Álvarez et al. 2023). *Proteobacteria* was the dominant type in both cases, making up 91.5% of bacteria present in these tissues, of which *Gammaproteobacteria* dominated. However, the addition of herbicide in the form of HIL resulted in a significant differentiation of the endophytes of both populations. *Actinobacteria* dominated (56% and 73.5%, respectively) in both the *S* and *R* weeds. Additionally, in the sensitive population, the second dominant type was *Proteobacteria* (43.5%), while in the resistant population, it was *Firmicutes* (25%). In terms of class contributions in these microbiomes, *Actinobacteria* (56%) and *Alphaproteobacteria* (43%) dominated in the sensitive plant, while in the resistant biotype, *Actinobacteria* accounted for 73%, while *Bacilli* constituted 25%. It can be clearly seen that the HIL compound had a significant impact on the composition of the microbiome of the examined plants; namely, the core microbiome remained the same as in untreated plants, but ionic liquid treatment resulted in an unexpected spike in the population of *Actinobacteria.*

#### Evaluation of biodiversity in soil microbiomes

The analysis of diversity parameters of bacterial communities revealed several key patterns in the plant microbiomes (Table 2). Firstly, it was observed that the root microbiome exhibited higher biodiversity compared to the shoot microbiome; furthermore, the biodiversity of microorganisms on the surfaces of shoot and roots surpassed that within their respective tissues. In the case of the phyllosphere, this phenomenon is caused by the fact that leaf surfaces are relatively lacking in nutrients in comparison to the rhizosphere and endosphere. Additionally, variations in heat, moisture and radiation throughout the day and seasons naturally result in lower biodiversity (Dastogeer et al. 2020). Additionally, Wagner et al. (2015) demonstrated that host plant genetic control of the microbiome is evident in leaves but not in roots (Wagner et al. 2016). Consequently, in this study, root endophyte biodiversity was more evident than shoot endophyte diversity.

**Table 2:** Biodiversity parameters of microbiome isolated from roots and shoots and foliage from susceptible and resistant biotypes of cornflower

		Biodiversity	Susceptible biotype			Resistant biotype		
			Control	Herbicide	HILs	Control	Herbicide	HILs
Roots	Epiphytes	OTU (operational taxonomic unit)	2039	1211	1511	1816	1383	1509
		Chao1bias	2639	1372	1613	2146	1284	2024
		Shannon index	6.15	5.67	5.75	5.93	5.79	5.69
		Phylogenetic diversity	5.32	4.92	4.9	5.21	5.06	5.11
	Endophytes	OTU (operational taxonomic unit)	1576	992	1087	1302	940	1312
		Chao1bias	1860	1325	1422	1525	1284	1470
		Shannon index	5.76	4.29	5.21	4.6	4.34	4.59
		Phylogenetic diversity	6.04	5.97	5.58	6.19	6.28	5.51
Shots and foliage	Epiphytes	OTU (operational taxonomic unit)	1702	1740	1426	1562	994	1462
		Chao1bias	1784	2217	1608	1320	1175	1845
		Shannon index	5.99	5.82	5.23	5.87	3.33	5.23
		Phylogenetic diversity	4.24	4.78	4.42	4.92	4.96	5.04
	Endophytes	OTU (operational taxonomic unit)	1445	938	813	1427	837	518
		Chao1bias	1613	1029	899	1544	887	581
		Shannon index	5.02	4.84	3.88	5.44	4.7	4.22
		Phylogenetic diversity	6.28	6.27	5.79	5.27	4.3	5.91

The microbiome of untreated plants exhibited greater biodiversity than that of plants subjected to herbicidal treatment. Such an outcome is to be expected; awareness of the impact of herbicides on the soil microbiome is growing, and alterations in the soil microbiome are assumed to impact crucial nutrient cycling and processes between plants and soil (Ruuskanen et al. 2023). However, no significant differences were observed in the root epiphyte microbiome of both susceptible and resistant weeds. Distinct variations were noticed in the shoot epiphyte microbiome in both plant types, which is probably caused by the specific properties of the phyllosphere mentioned above (Dastogeer et al. 2020).

### Gene presence in biological samples

Following the assessment of the impact of the herbicide and HIL on the structure of the microbiome of sensitive and resistant biotypes of cornflower, we have attempted to determine the presence of genes that encode enzymes involved in the catabolism of sulfonylurea herbicides in plant surface and tissues as well as rhizosphere. The selected genes originate from soil-borne bacteria and have been proven to participate in the transformation of sulfonylurea herbicides into biologically inactive forms (Hang et al. 2012; Harder et al. 1991; Hussain and Ward 2003; Kang et al. 2014, 2012; Lu et al. 2012; Omer et al. 1990; H. Zhang et al. 2018a, b; Z. Zhang et al. 2018a, b). Moreover, evidence suggests that bacteria with the ability to produce enzymes that detoxify iodosulfuron may enable other bacteria susceptible to sulfonylurea herbicides to grow in a sulfonylurea-contaminated environment (Arabet et al. 2014). Thus, they either promote the spread of genes encoding resistance to herbicides or induce the evolution of resistance in susceptible bacteria when the minimal lethal concentration is not reached.

*B. subtilis* YB1, initially discovered in Chinese farmlands polluted with sulfonylureas, demonstrated the capability to degrade up to 80% of nicosulfuron in liquid medium (Lu et al. 2012), and was proven to utilise nicosulfuron as a sole carbon source under aerobic conditions (Kang et al. 2012). Further analyses in a chamber study confirmed its efficacy in degrading a wide array of herbicides from this group, including rimsulfuron, bensulfuron methyl, pyrazosulfuron-ethyl, cinosulfuron and tribenuron-methyl (Zhang et al. 2020). Crucially, the enzymes involved in the degradation, namely manganese ABC transporter, vegetative catalase 1 and acetoin dehydrogenase E1 (Kang et al. 2014; Z. Zhang et al. 2018a, b), are extracellular proteins that have been shown to be expressed after induction (Kang et al. 2014, 2012), and act via pyrimidine ring and sulfonylurea bridge cleavage (Z. Zhang et al. 2018a, b). Unfortunately, we were unable to obtain strain YB1; hence, the model strain *B. subtilis* 168 was used instead of *B. subtilis* YB1. However, the amino acid sequence of *B. subtilis* YB1 matches that of *B. subtilis* 168 very closely; the manganese ABC transporter sequence is 99% identical, vegetative catalase 1 is a 100% match and the acetoin dehydrogenase amino acid sequence matches by 99% (Kang et al. 2014; Z. Zhang et al. 2018a, b).

Another bacterium with sulfonylurea-degrading ability utilised in this study is *S. griseolus* 14,576–4 (also known as *S. halstedii*) (Harder et al. 1991; Omer et al. 1990). *S. griseolus* can metabolize various sulfonylurea herbicides into often less phytotoxic compounds, which is facilitated by two sulfonylurea-inducible cytochrome P-450 monooxygenases: cytochrome P-450_SU-1_ and cytochrome P-450_SU-2_ (Omer et al. 1990). Partial characterization and reconstitution studies suggest that the two inducible P-450 monooxygenase systems in *S. griseolus* share similarities with the three-component cytochrome P-450_CAM_ camphor oxidation system found in *P. putida* (Harder et al. 1991; Omer et al. 1990).

Another bacterium utilized in this study, *H. zhihuiae* S133, was isolated from heavily sulfonylurea herbicide-contaminated farmland soil in Jiangsu province, China (Wen et al. 2011). It is capable of producing a constitutively expressed deestrification estrase, *SulE*, which can transform thifensulfuron-methyl, metsulfuron-methyl, bensulfuron-methyl, ethametsulfuron-methyl and chlorimuron-ethyl into herbicidally inactive corresponding acids forms (Hang et al. 2012; Liu et al. 2019). Furthermore, it has been shown that this bacterium can survive in the rhizosphere of cucumber, colonize its roots and efficiently degrade chlorimuron-ethyl in the plants’ rhizosphere (H. Zhang et al. 2018a, b). In addition, *H. zhihuiae* CHL1, with 98% similarity of 16S rRNA gene sequence to *H. zhihuiae* S133, was isolated from soil by (Yang et al. 2014). This strain efficiently transforms chlorimuron-ethyl, metsulfuron-methyl and tribenuron-methyl (Yang et al. 2014). Therefore, the dissemination of *SulE* enzyme may be more widespread in the soil environment than initially assumed.

#### Soil and rhizosphere

All investigated genes encoding the aforementioned enzymes, except for vegetative catalase from *B. subtilis*, were found in the samples isolated from soil (Table 3). In case of acetoin dehydrogenase-encoding genes, no discernible trend was immediately visible, as both primers targeting this enzyme’s gene were present at similar rates in all samples isolated from both susceptible and resistant cultivars’ soil. Regarding the manganese ABC transporter, the cytochrome P450 enzyme genes appeared to be more prevalent in the soil with the resistant biotype. As for the sulfonylurea deestrification estrase *SulE* encoding gene, it was primarily found in both cultivars in samples exposed to HIL rather than the herbicide, although it could also be detected in the soil of untreated resistant weeds.

**Table 3:**
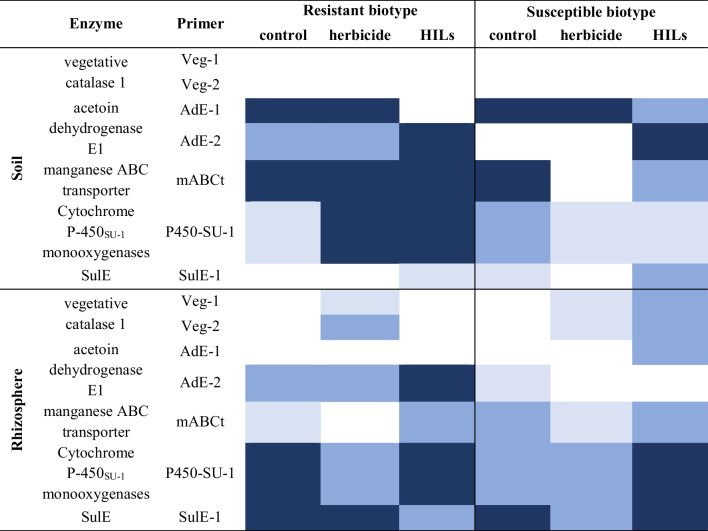
Heat-map representing the frequency of occurrence of genes associated with ISM biotransformation in microbiomes isolated from soil and rhizosphere. Dark blue – gene in 3 biological replicates, blue – gene in 2 biological replicates, light blue – gene in 1 biological replicate, lack of colour indicate no gene presence was detected

A single general trend was observed in the case of rhizosphere, namely, a decrease in the presence of genes derived from *B. subtilis* in control samples and samples exposed to herbicidal treatment, while in the susceptible population, the previously found genes encoding *B. subtilis* enzymes and vegetative catalase were present. Moreover, an increase of the presence of both P450 SU-1 and SulE encoding genes could be observed. The latter result is particularly notable, as it has been proven *H. zhihuaiae* is capable of penetrating plant tissues and persisting as an endophyte (H. Zhang et al. 2018a, b). Additionally, it has been evidenced that root secretions promote the growth of this bacterium.

#### Root epiphytes and endophytes

Generally, the number of gene copies in both cornflower populations in root samples appears lower than in the case of rhizosphere (Table 4). Notably, more gene copies can be observed in the susceptible cultivar compared to the resistant one. However, of the greatest importance is the fact that the *SulE* gene can be observed in both epi- and endophytes of susceptible cultivar treated with HILs, and in epiphytes in the case of commercial herbicide treatment. In contrast, in the resistant cultivar, *SulE* genes were present only in control sample’s epiphytes. According to Zhang et. al. (2018), *H. zhihuaiae* can biotransform and thus detoxify sulfonylurea herbicides in the plant rhizosphere (H. Zhang et al. 2018a, b). Additionally, primers targeting the *SulE* gene have been used as a tool-marker to isolate new strains of *Hanschlegiella* bacteria (Yang et al. 2014).

**Table 4:**
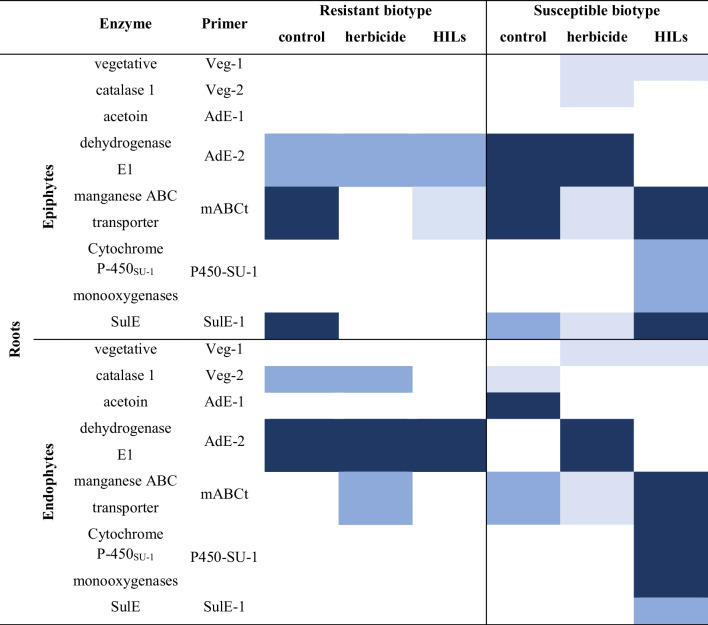
Heat-map representing the frequency of occurrence of genes associated with ISM biotransformation in microbiomes isolated from root surface and root tissue. Dark blue – gene in 3 biological replicates, blue – gene in 2 biological replicates, light blue – gene in 1 biological replicate, lack of colour indicate no gene presence was detected

The acetoin dehydrogenase-encoding gene and the gene encoding manganese ABC transporter were present in samples isolated both from epiphytes and endophytes in root samples of susceptible and resistant cornflower. However, vegetative catalase was more commonly found in S biotype than in the R biotype. Notably, the cytochrome P450 enzyme encoding-gene was found only in the susceptible population treated with HILs.

#### Shoot epiphytes and endophytes

Samples isolated from shoots and foliage were analysed (Table 5). It appears that genes encoding enzymes participating in sulfonylurea degradation were less commonly found in the *S* biotype compared to the R biotypes. Vegetative catalase, acetoin dehydrogenase, manganese ABC transporter and sulfonylurea esterase-encoding genes were found in epiphytic samples of both susceptible and resistant populations treated with the herbicide. However, after treatment with HILs, these genes were only found in resistant plants. On the other hand, all genes encoding enzymes conferring resistance to sulfonylureas were found in endophytic samples isolated from the resistant biotype, while only SulE and P450 were found in the susceptible biotype. The acetoin dehydrogenase gene was also detected in resistant cornflower treated with HILs compared to the same population treated with the sole herbicide.

**Table 5:**
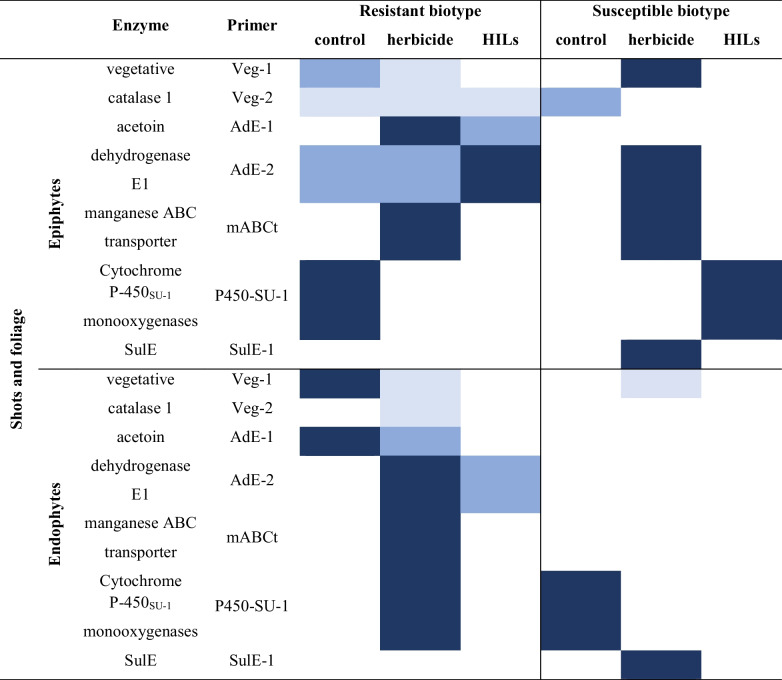
Heat-map representing the frequency of occurrence of genes associated with ISM biotransformation in microbiomes isolated from shoots and foliage. Dark blue – gene in 3 biological replicates, blue – gene in 2 biological replicates, light blue – gene in 1 biological replicate, lack of colour indicate no gene presence was detected

The analysis of gene presence in environmental samples of soil and plant tissues revealed an important aspect associated with the genes encoding enzymes that facilitate bacterial degradation of sulfonylureas, namely that genes originating from *B. subtilis*, *S. griseolus* and especially *H. zhihuaiae* were more widespread in samples treated with herbicide and HILs than initially suspected. They were the most common in soil and rhizosphere, with slightly higher prevalence in samples in which the resistant biotype of cornflower was growing. Higher diversity of genes was generally observed in the resistant biotype samples derived from the shoots and foliage; in contrast, this trend was reversed in case of samples derived from plant roots as the susceptible biotype was characterized by a broader spectrum of determined genes. Moreover, no conclusive observations regarding the impact of the HILs and herbicide on the gene frequency could be made.

### Gene abundance in biological samples

In order to provide further insight regarding genes associated with degradation of sulfonylurea herbicides, the final stage of studies was focused on the assessment of their abundance using RT-qPCR technique in the microbiomes originating from the surface and inner tissues of roots and shoot obtained from both susceptible and resistant biotypes, using 16S RNA gene as a reference.

**Fig. 5:**
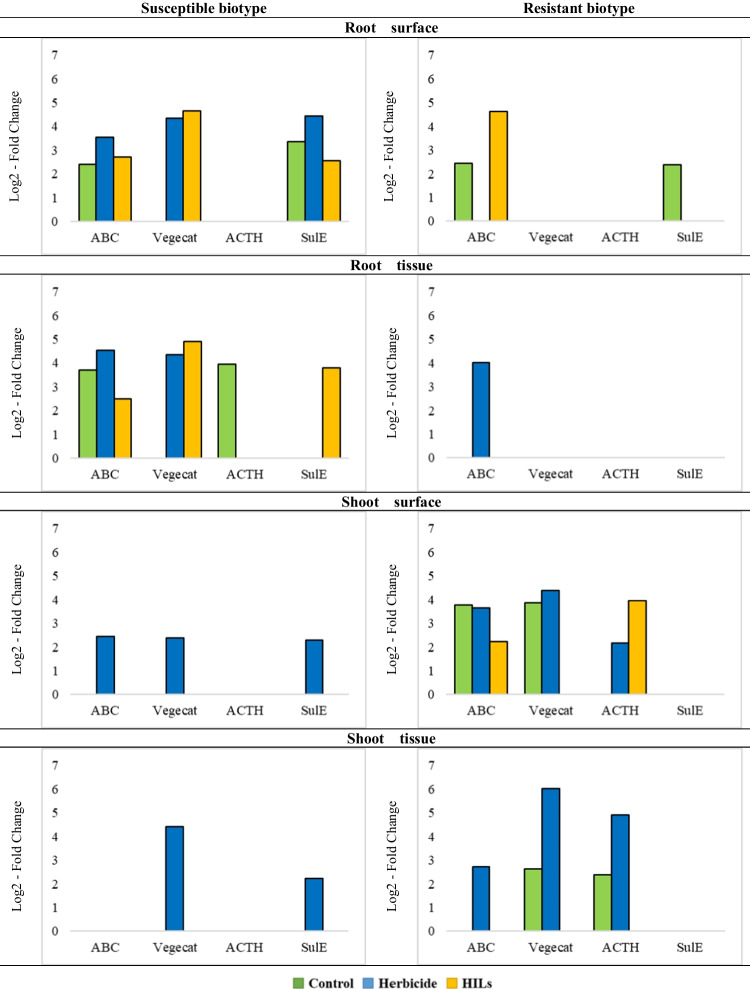
Log2-fold change values determined by real-time PCR of ABC, Vegecat, ACTH and SulE genes from samples isolated from resistant and susceptible cultivars of cornflower. Green indicates control group, blue indicates group of plants sprayed with herbicide and yellow indicates group of plants sprayed with HILs

The Cytochrome P-450_SU-1_ genes, which were determined as present in shoot samples of both susceptible and resistant biotypes as well as in root samples of resistant biotypes, could not be efficiently quantified via RT-qPCR (Fig. 5 and Table S1). This finding indicates that, although the genes are present, their abundance in samples is low.

The root surface of the susceptible biotype has exhibited a higher enrichment in the examined genes compared to the root surface of the resistant biotype. This phenomenon might be attributed to the fact that resistant plants are inherently better adapted to biotransform sulfonylureas utilising molecular mechanisms present in their tissues, rather than relying on external factors, such as microbes. In contrast, susceptible plants, lacking these molecular mechanisms, are more likely to cope with the presence of xenobiotics with the assistance of specialised bacteria possessing genes participating in herbicide degradation (Tétard-Jones and Edwards 2016). The acetoin dehydrogenase gene was not observed in either weed biotype, and the vegetative catalase gene was absent in resistant cornflower. However, the vegetative catalase gene was present in the susceptible population, with slightly higher number of genes found in HIL-treated plants compared to herbicide-treated plants, while the gene could not be quantified in control plants. These findings correspond well with the results described in section ‘Root surfaces’, as *Firmicutes* were characterized by the second highest abundance in the microbiome of HIL- and herbicide-treated susceptible plants, whereas the lowest *Firmicutes* ratio was observed in the control sample (which was lower by one order of magnitude compared to treated samples). Moreover, genes encoding manganese ABC transporter were found in all treatments of sensitive cornflower, with up to a twofold higher abundance in HIL-treated resistant biotype. For the SulE de-esterification esterase genes, they were present in all treatments of the sensitive biotype, with the highest abundance in herbicide-treated samples. Based on these results, it is challenging to determine the impact of herbicide form (ionic liquid or pure herbicide) on the abundance of genes encoding enzymes degrading sulfonylureas, but a clear trend shows these genes are more frequent in the microbiome of plants subjected to either treatment than in the control.

In the inner parts of roots, only manganese ABC transporter could be quantified in the resistant population, suggesting that the abundance of other genes was too low to be detected despite being identified in previous analyses. Intriguingly, vegetative catalase was detected at similar levels in susceptible plants’ roots as in the rhizosphere, despite a significant shift in microbial community composition. This phenomenon is even more evident for manganese ABC transporter, as it was the least abundant in HILs treatment, despite *Firmicutes* making up 54% of the community, whereas in case of herbicide treatment it was twofold higher, despite *Firmicutes* constituting less than 5% of the isolated microbiome.

The shoot surface and inner tissues of the *S* biotype were similar to the root tissue of *R* biotype, with quantifiable genes only in herbicidal treatment, despite some being detected in previous analyses, suggesting their scarcity in the microcosm. On the other hand, a significantly higher number of gene copies encoding vegetative catalase and acetoin dehydrogenase were detected in resistant weeds after herbicide application than in the untreated population, suggesting bacteria may aid the plant in detoxifying this herbicide. The absence of these genes in samples subjected to HIL treatment implies that cation toxicity impacts the inner tissue microbiome, aligning well with recent trends in utilizing HILs as potential tools for controlling herbicide-resistant weeds (Marcinkowska et al. 2023; Pernak et al. 2022).

## Conclusions

Initial evaluation of herbicidal efficacy against cornflower revealed that the herbicide and HIL exhibit comparable activity, which was satisfactory (> 85%) in case of susceptible biotypes and low (< 15%) for resistant biotypes. On the basis of these results, it can be established that HIL based on a single herbicide are not effective for control of herbicide resistant weeds; thus, the previously mentioned necessity to employ several active compounds seems justified. However, subsequent genetic analyses elucidate additional aspects: (i) treatment with HIL results in significant shifts in bacterial microbiomes, as evidenced by a higher ratio of *Actinobacteria* in shoot inner tissue for both biotypes as well as increased abundance of *Firmicutes* in root and shoot endophytes of the resistant biotype; (ii) the presence of the herbicide or HIL leads to decreased microbial alpha-diversity (reflected by decreased OUTs, Chao1 and Shannon indices) relative to control, and the effect of HIL was particularly visible in case of shoot endophytes; (iii) application of HIL decreased the occurrence and abundance of genes associated with the degradation of sulfonylurea herbicides in root and shoot inner tissues of resistant biotypes compared to both herbicide-treated samples and untreated control.

The abovementioned findings contribute to the novelty and significance of the study, as they highlight the potential of HILs to hinder microbiome conferred herbicide resistance in weeds. On the other hand, the obtained results also indicate that the impact of HILs on the soil microbiome is higher compared to classic herbicides, which should be included as an additional factor in risk assessment during the registration of such compounds.

## Supplementary Information

Below is the link to the electronic supplementary material.

Supplementary file1 (DOCX 364 KB)

## Data Availability

Data will be made available on reasonable request.
