# Supplementary material for: Comparative analysis of bacterial populations in sulfonylurea-sensitive and -resistant weeds: insights into community composition and catabolic gene dynamics
Source: Environ Sci Pollut Res Int. 2024 Aug 16;31(39):52391–409. doi: 10.1007/s11356-024-34593-z (PMC11374828; doi:10.1007/s11356-024-34593-z)
Supplement: Supplementary file 1 — Supplementary file1 (DOCX 364 KB) [file 11356_2024_34593_MOESM1_ESM.docx]

**Comparative Analysis of Bacterial Populations in Sulfonylurea-Sensitive and -Resistant Weeds: Insights into Community Composition and Catabolic Gene Dynamics**

Jan Homa^a*^, Wiktoria Wilms^a,b^, Katarzyna Marcinkowska^c^, Paweł Cyplik^d^, Łukasz Ławniczak^a^, Marta Woźniak-Karczewska^a^, Michał Niemczak^a^, Łukasz Chrzanowski^a^

^a^Department of Chemical Technology, Poznan University of Technology, 60-965 Poznan, Poland

^b^Institute for Environmental Studies, Faculty of Science, Charles University in Prague, 128 01 Prague, Czech Republic

^c^Department of Weed Science, Institute of Plant Protection – National Research Institute, 60-318 Poznan, Poland

^d^Department of Food Technology of Plant Origin, Poznan University of Life Sciences, 60-624 Poznan, Poland

*corresponding author: jan.j.homa@doctorate.put.poznan.pl; Department of Chemical Technology, Poznan University of Technology, Berdychowo 4, 60-965 Poznań, Poland

**Table S1** Genes, primer names, and primer sequences used for PCR analysis.

| **Gene name** | **Primer name** | **Primer Sequence** | **Ref.** |
| --- | --- | --- | --- |
| sulE | sulEF1 | 5’-CTGATTGCATATGGAAACTGACAACGTGGAGCT-3’ | [1] |
|  | sulER1 | 5’-TACAAGCTTGCTTTCGTTCTGATCTAAGC-3’ |  |
| vegetative  catalase 1 | veg2F | 5’-GGAATTCATGAGTTCAAATAAACTGACAAC-3’ |  |
|  | veg2R | 5’-CCGCTCGAGTTAAGAATCTTTTTTAATCGGC-3’; |  |
| Acetoin  dehydrogenase E1 | adE2F | 5’-CGGGATCCATGGCGAGAGTCATAAGCATGTC-3’ |  |
|  | adE2R | 5’-CCGCTCGAGTTAATTCAATGCCGGCTCGC-3’ |  |
| manganese  ABC transporter | mABCtF | 5’-CATGCATATGAAAAGCGCTGATCAGCAA-3’ | [2] |
|  | mABCtR | 5’-CATGCTCGAGTTATTTAGTAATCG-3’ |  |
| Vegetative  catalase 1 | veg1F | 5’-CATGCATATGAGTTCAAATAAACTGACA-3’ |  |
|  | veg1R | 5’-CATGCTCGAGTTAAGAATCTTTTTTAATCGGCAA-3’ |  |
| acetoin dehydrogenase E1 | adE1F | 5’-CATGCATATGGCGAGAGTCATAAGC-3’ |  |
|  | adE1R | 5’-CATGCTCGAGTTAGGCTCGC-3’ |  |
| P450SU1 and its adjacent ferredoxin, Fd1 (CYP 105A1 gene) | P450SU1-Fd1F | 5’-GTCATATGACCGATACCGCCACGACG-3’ | [3] |
|  | P450SU1-Fd1R | 5’-CTGGATCCTATTCCGTGTCCTCGACG-3’ |  |

**Table S2** Primers and corresponding temperatures optimised for PCR use.

| **Primer pair name** | **Temperature [°C]** |
| --- | --- |
| Sul E1 | 56 |
| mABCt-1 | 65 |
| Veg-1 | 58 |
| Veg-2 | 56 |
| AdE1 | 58 |
| adE2 | 65 |
| P450 SU-1 | 65 |

**Table S3** Genes, primer names, and primer sequences used for qPCR analysis.

| **Target Gene** | **Primer name** | **Forward sequence** | **Reverse sequence** |
| --- | --- | --- | --- |
| sulE | Hz-sulE | 5’-CGACGGCCTGAAAAGAGGAT-3’ | 5’-AGGAACAGAGGGCCGATACT-3’ |
| manganese  ABC transporter | Bs-ABC | 5’-CTGCTGCATCGGCAATTTGA-3’ | 5’-TGGCAGCAGTACTTTTTGCG-3’ |
| Vegetative  catalase 1 | Bs-vegcat | 5’-CGATTTGGCGCAGCTTGATT-3’ | 5’-TCAAGCCGGCGATCTGTATC-3’ |
| acetoin dehydrogenase E1 | Bs-Actdh | 5’-GAAAGAGCAAGAAACGGCGG-3’ | 5’-AGGTGCTCAACTCTTTCATCC-3’ |
| 16 S rRNA | F968 Forward R1401 Reverse | 5’-AACGCGAAGAACCTTAC-3’ | 5’-CGGTGTGTACAAGACCC-3’ |

**
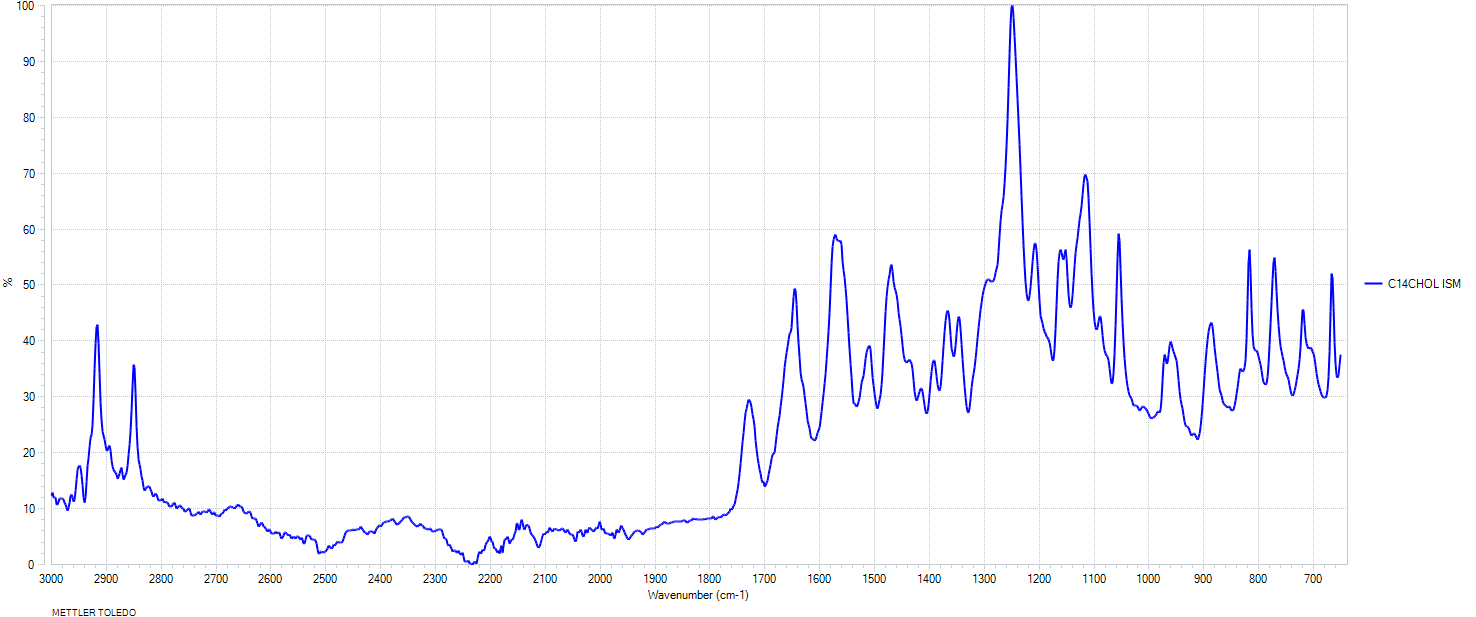
**

IR υ_max_ [cm^-1^] = 2953, 2915, 2850, 1729, 1646, 1571, 1510, 1468, 1392, 1366, 1348, 1249, 1206, 1161, 1152, 1114, 1090, 1055, 971, 960, 885, 816, 772, 720, 667.

**Fig. S1** FTIR spectrum of N-tetradecylcholine iodosulfuron-methyl [C_14_CHOL][ISM].


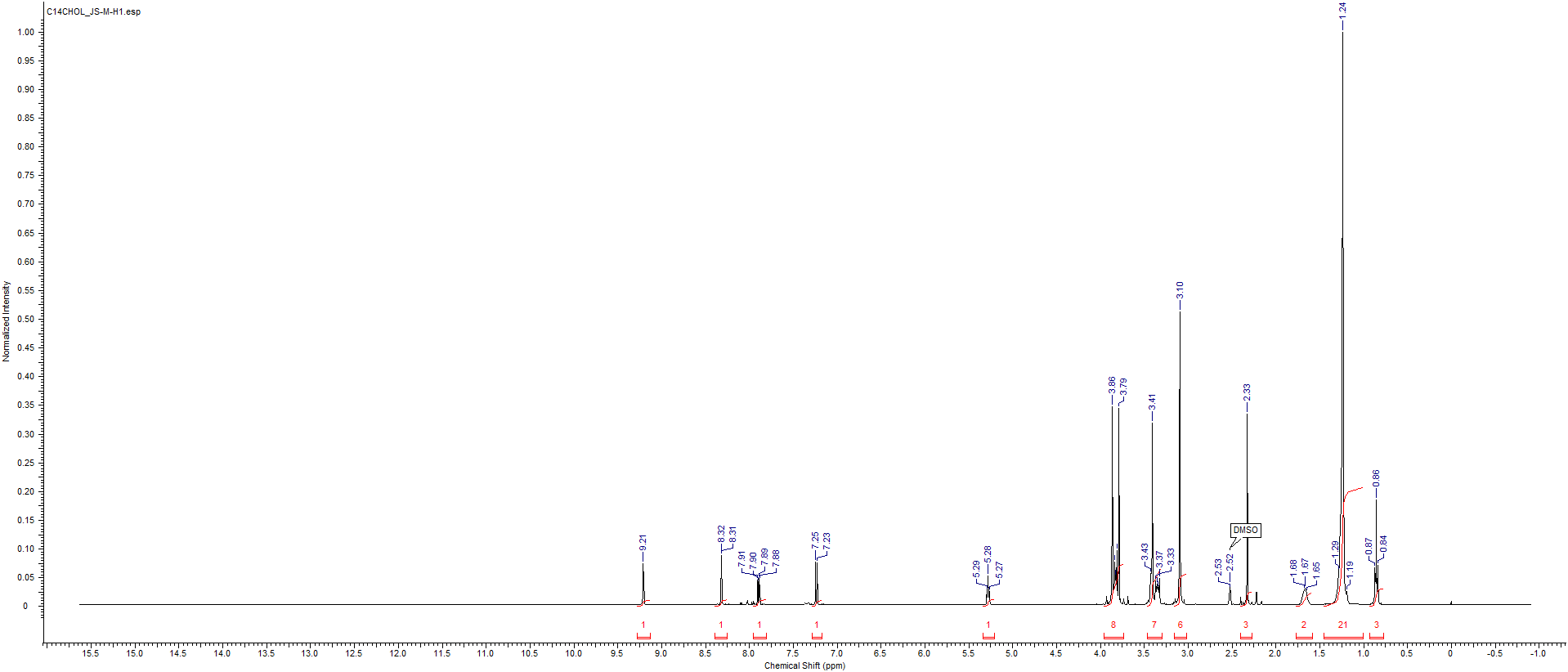


^1^H NMR (400 MHz, DMSO-*d_6_*) δ [ppm] = 0.86 (3H, t, *J* = 6.8 Hz); 1.24 (21H, m); 1.67 (2H, m); 2.33 (3H, s); 3.10 (6H, s); 3.41 (7H, m); 3.83 (8H, m); 5.28 (1H, m); 7.24 (1H, d, *J* = 8.1 Hz); 7.90 (1H, dd, *J_12_* = 8.0 Hz, *J_13_* = 1.7 Hz); 8.31 (1H, d, *J* = 1.8 Hz), 9.21 (1H, s).

**Fig. S2** ^1^H NMR spectrum of N-tetradecylcholine iodosulfuron-methyl [C_14_CHOL][ISM].


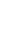


^13^C NMR (100 MHz, CD_3_OD) δ [ppm] = 13.9, 21.8, 22.1, 25.1, 25.8, 28.7, 29.0, 31.3, 50.8, 52.7, 54.3, 54.9, 64.1, 64.5, 96.3, 129.3, 130.8, 137.3; 138.6, 144.4, 155.0, 165.5, 168.3, 170.6, 177.7.


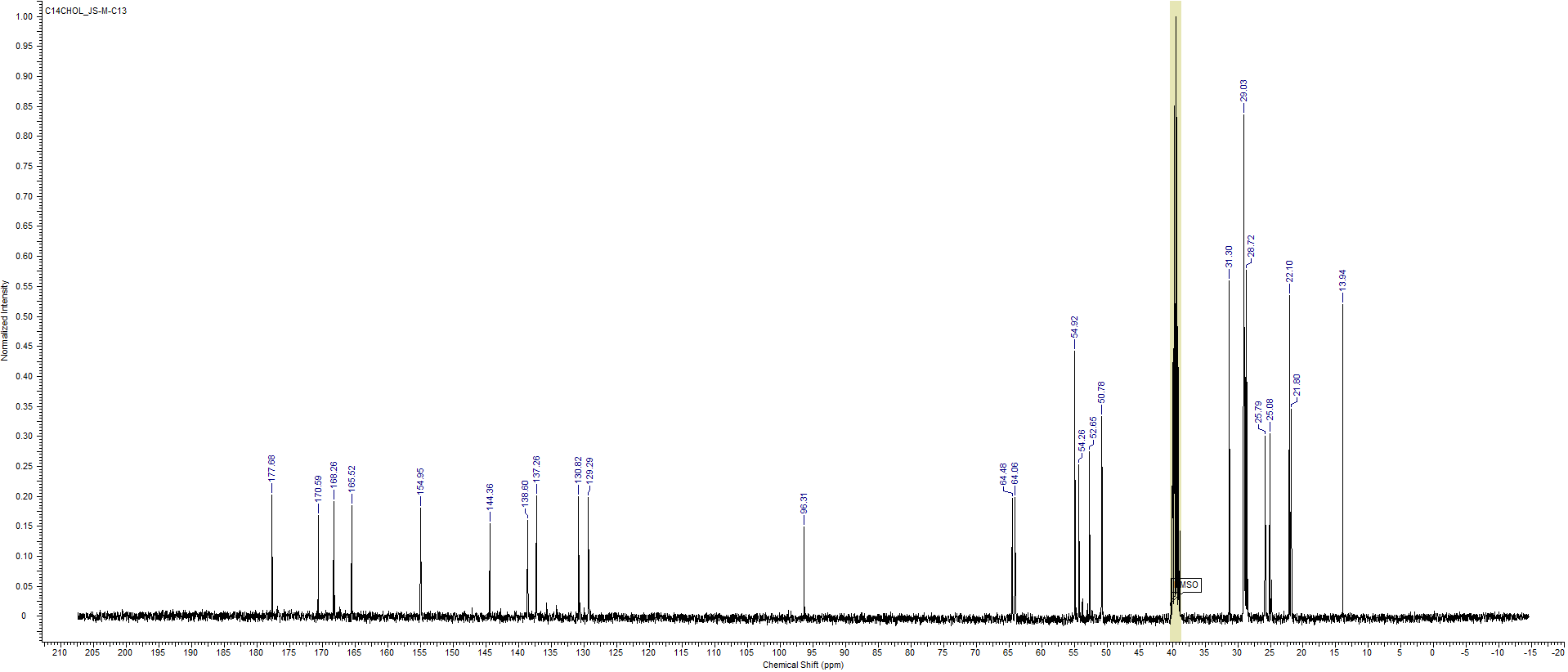


**Fig. S3** ^13^C NMR spectrum of N-tetradecylcholine iodosulfuron-methyl [C_14_CHOL][ISM].

**Fig. S4** The influence of the tested HILs and reference herbicide on the cornflower biotypes resistant (R) and sensitive (S) to ALS inhibitors.

**Fig. S5** Analysis of the root surface microbiome composition of susceptible and herbicide-resistant cornflower. C – untreated control; H – herbicide treatment; HILs – herbicidal ionic liquid treatment.

**Table S4** C_T_ target/C_T_ 16 S values and SEM values obtained from qPCR analysis of gene abundance in the environment.

| **Suscceptible Biotype** | | | | |
| --- | --- | --- | --- | --- |
| **Root Surface** | ABC | Vegecat | ACTH | SulE |
| Control | 2.40 ± 0.2 | - | - | 3.36 ± 0.16 |
| Herbicide | 3.54 ± 0.36 | 4.36 ± 0.35 | - | 4.46 ± 0,45 |
| HILs | 2.70 ± 0.02 | 4.64 ± 0.16 | - | 2.55 ± 0.12 |
| **Root Tissue** | ABC | Vegecat | ACTH | SulE |
| Control | 3.70 ± 0.07 | - | 3.94 ± 0.11 | - |
| Herbicide | 4.52 ± 0.2 | 4.36 ± 0.18 | - |  |
| HILs | 2.50 ± 0.11 | 4.89 ± 0.09 | - | 3.80 ± 0.23 |
| **Stem Surface** | ABC | Vegecat | ACTH | SulE |
| Control | - | - | - | - |
| Herbicide | 2.46 ± 0.11 | 2.39 ± 0.12 | - | 2.30 ± 0.18 |
| HILs | - | - | - | - |
| **Stem Tissue** | ABC | Vegecat | ACTH | SulE |
| Control | - | - | - | - |
| Herbicide | - | 4.41 ± 0.18 | - | 2.27 ± 0.13 |
| HILs | - | - | - | - |
| **Resistant Biotype** | | | | |
| **Root Surface** | ABC | Vegecat | ACTH | SulE |
| Control | 2.45 ± 0.08 | - | - | 2.37 ± 0.12 |
| Herbicide | - | - | - | - |
| HILs | 4.64 ± 0.07 | - | - | - |
| **Root Tissue** | ABC | Vegecat | ACTH | SulE |
| Control | - | - | - | - |
| Herbicide | 4.00 ±0.26 | - | - | - |
| HILs | - | - | - | - |
| **Stem Surface** | ABC | Vegecat | ACTH | SulE |
| Control | 3.76 ± 0.13 | 3.87 ± 0.07 | - | - |
| Herbicide | 3.64 ± 0.17 | 4.40 ± 0.12 | 2.15 ±0.03 | - |
| HILs | 2.24 ± 0.13 | - | 3.96 ±0.07 | - |
| **Stem Tissue** | ABC | Vegecat | ACTH | SulE |
| Control | - | 2.63 ±0.05 | 2.39 ±0.08 | - |
| Herbicide | 2.72 ±0.12 | 6.03 ±0.43 | 4.91 ±0.3 | - |
| HILs | - | - | - | - |

**References:**

[1] B.J. Hang, Q. Hong, X.T. Xie, X. Huang, C.H. Wang, J. He, S.P. Li, SulE, a sulfonylurea herbicide de-esterification esterase from Hansschlegelia zhihuaiae S113, Appl. Environ. Microbiol. 78 (2012) 1962–1968. https://doi.org/10.1128/AEM.07440-11.

[2] Z. Zhang, Y. Zhang, D.C. Yang, J.L. Zhang, Expression and functional analysis of three nicosulfuron-degrading enzymes from Bacillus subtilis YB1, J. Environ. Sci. Heal. - Part B Pestic. Food Contam. Agric. Wastes. 53 (2018) 476–485. https://doi.org/10.1080/03601234.2018.1455344.

[3] H.A. Hussain, J.M. Ward, Enhanced heterologous expression of two Streptomyces griseolus cytochrome P450s and Streptomyces coelicolor ferredoxin reductase as potentially efficient hydroxylation catalysts, Appl. Environ. Microbiol. 69 (2003) 373–382. https://doi.org/10.1128/AEM.69.1.373-382.2003.
